# Possible Transmission of *mcr-1*–Harboring *Escherichia coli* between Companion Animals and Human

**DOI:** 10.3201/eid2209.160464

**Published:** 2016-09

**Authors:** Xue-Fei Zhang, Yohei Doi, Xi Huang, Hong-Yu Li, Lan-Lan Zhong, Kun-Jiao Zeng, Yan-Fen Zhang, Sandip Patil, Guo-Bao Tian

**Affiliations:** Sun Yat-Sen University Zhongshan School of Medicine, Guangzhou, China (X.-.F Zhang, X. Huang, L.-L. Zhong, K.-J. Zeng, Y.-F. Zhang, S. Patil, G.-B. Tian);; Ministry of Education Key Laboratory of Tropical Diseases Control, Guangzhou (X.-F. Zhang, X. Huang, L.-L. Zhong, K.-J. Zeng, Y.-F. Zhang, S. Patil, G.-B. Tian);; University of Pittsburgh Medical Center, Pittsburgh, Pennsylvania, USA (Y. Doi);; Sun Yat-Sen University Memorial Hospital, Guangzhou (H.-Y. Li)

**Keywords:** colistin, mcr-1, companion animals, pets, dogs, cats, Escherichia coli, enterobacteriaceae, bacteria

**To the Editor:** Plasmid-mediated, colistin-resistance mechanism gene *mcr-1* was first identified in *Escherichia coli* isolates from food, food animals, and human patients in November 2015 (*1*). Reports on detection of *mcr-1* in *Enterobacteriaceae* from humans and food animals soon followed from ≈12 countries (*2*–*5*). Here we report detection of *mcr-1* in colistin-resistant *E. coli* isolated from companion animals and the possible transmission of *mcr-1*–harboring *E. coli* between companion animals and a person.

Three *mcr-1*–harboring *E. coli* clinical isolates were identified from specimens of 3 patients admitted to a urology ward of a hospital in Guangzhou, China. *E. coli* isolate EC07 was identified in the urine of a 50-year-old male patient with glomerulonephritis in October 2015. Isolate EC08 was cultured from the urine of a 48-year-old male patient with prostatitis in December 2015. Isolate EC09 was identified in the blood of an 80-year-old male patient with bladder cancer 3 weeks after EC08 was identified.

Review of medical records identified the patient carrying *E. coli* isolate EC07 as a worker at a pet shop. In light of this finding, we collected a total of 53 fecal samples from 39 dogs and 14 cats in the pet shop where the man worked. We isolated and identified colonies consistent with *E. coli* from fecal samples on MacConkey agar plates (Thermo Fisher, Beijing, China) and API 20E system (bioMérieux, Durham, NC, USA). We prepared crude DNA samples of isolates for PCR testing by boiling cells in water. Among them, 6 were positive for *mcr-1* by PCR and sequencing (4 from dogs and 2 from cats). All 6 isolates were resistant to colistin, polymyxin B, cephalosporin, gentamicin, and ciprofloxacin by using the agar dilution method, in accordance with the European Committee on Antimicrobial Susceptibility Testing (http://www.eucast.org) for colistin and polymyxin B and Clinical and Laboratory Standards Institute guidelines (http://www.clsi.org) for the other antimicrobial drugs. We identified various resistance genes accounting for the multidrug resistance in these 9 *mcr-1–*positive isolates (*6*,*7*) (Table). We noted that *E. coli* isolate EC09 was also resistant to carbapenems and positive for *bla*_IMP-4_. We observed co-production of *mcr-1* and IMP-type metallo-β-lactamase in *E. coli*.

**Table:** Characteristics of 9 *mcr-1*–positive *Escherichia coli* isolates from companion animals and human patients, Guangzhou, China

Characteristic	Isolate								
	PET01	PET02	PET03	PET04	PET05	PET06	EC07	EC08	EC09
Isolation date	2016 Jan 1	2016 Jan 1	2016 Jan 2	2016 Jan 2	2016 Jan 2	2016 Jan 4	2015 Oct 10	2015 Nov 2	2015 Nov 21
Specimen source	Cat	Dog	Dog	Dog	Cat	Dog	Human	Human	Human
Specimen type	Feces	Feces	Feces	Feces	Feces	Feces	Urine	Urine	Blood
Phylogenetic group	B2	D	D	D	B2	D	D	B1	B1
ST†	ST93	ST354	ST354	ST354	New	ST354	ST354	ST156	ST156
PFGE type	IV	I	I	I	V	I	I	II	III
Resistance genes	*mcr-1*, *bla*_TEM-1_, *qepA*	*mcr-1*, *bla*_TEM-1_, *bla*_CTX-M-15_, *fosA3, aac(6′)-Ib*-cr	*mcr-1*, *bla*_TEM-1_, *bla*_CTX-M-15_, *fosA3, aac(6′)-Ib*-cr	*mcr-1*, *bla*_TEM-1_, *bla*_CTX-M-15_, *fosA3, aac(6′)-Ib*-cr	*mcr-1*, *bla*_TEM-1_, *bla*_SHV-12_, *bla*_CTX-M-15_, *fosA3, rmtB, qnrS, aac(6′)-Ib*-cr	*mcr-1*, *bla*_TEM-1_, *bla*_CTX-M-15_, *fosA3, aac(6′)-Ib*-cr	*mcr-1*, *bla*_TEM-1_, *bla*_CTX-M-15_, *fosA3, aac(6′)-Ib*-cr	*mcr-1*, *bla*_TEM-1_, *bla*_CTX-M-55_, *fosA3, rmtB, qepA1*	*mcr-1*, *bla*_IMP-4_, *bla*_TEM-1_, *bla*_CTX-M-55_, *fosA3, rmtB, qepA1*
MIC, μg/mL									
Colistin	16	8	8	16	16	8	8	8	64
Polymyxin B	16	16	16	32	16	16	8	8	64
Ampicillin	>256	>256	>256	>256	>256	>256	>256	>256	>256
AMX/CLV	16	32	32	32	256	16	32	16	16
Cefotaxime	64	>256	256	>256	256	>256	256	256	>256
Ceftazidime	16	256	128	256	64	256	128	32	>256
Cefepime	8	256	128	256	16	256	64	64	>256
Gentamicin	128	>256	>256	>256	256	>256	>256	>256	>256
Amikacin	4	>256	>256	>256	>256	>256	>256	>256	>256
Ertapenem	<0.25	1	0.5	0.25	<0.25	1	0.5	<0.25	>16
Imipenem	<0.25	<0.25	<0.25	<0.25	<0.25	<0.25	<0.25	<0.25	>16
Meropenem	<0.25	<0.25	<0.25	<0.25	<0.25	<0.25	<0.25	<0.25	>16
Fosfomycin	32	>512	>512	>512	>512	>512	>512	>512	>512
Tigecycline	<2	<2	<2	<2	<2	<2	<2	<2	4
Nitrofurantoin	<16	32	32	32	128	32	64	64	32
Ciprofloxacin	256	128	128	128	64	128	128	256	256

*AMX/CLV, amoxicillin clavulanic acid; PFGE, pulsed-field gel electrophoresis; ST, sequence type. †By multilocus sequence typing.

We subjected all isolates to multilocus sequence typing, in accordance with the protocol described at http://mlst.warwick.ac.uk/mlst/dbs/Ecoli, and pulsed-field gel electrophoresis as described previously (*8*–*10*). We identified 5 *mcr-1–*positive isolates from 4 dogs (PET02–04 and PET06) and isolate EC07 as sequence type (ST) 354. Isolates PET01 and PET05, identified from cats, belonged to ST93 and a new ST strain, respectively. Isolates EC08 and EC09, from the patients who shared the same hospital room with the pet shop worker, were ST156 (Table). Results of pulsed-field gel electrophoresis were consistent with multilocus sequence typing results and showed that isolates consisted of 5 types (types I to V; Technical Appendix). Isolate EC07 was clonally related to 4 *E. coli* strains from dogs, according criteria described by Tenover et al. (*10*), suggesting possible transmission of *mcr-1*–harboring *E. coli* between dogs and the patient. Colistin resistance was successfully transferred to *E. coli* C600 through conjugation in all isolates, suggesting that *mcr-1* was located on transferable plasmids.

These findings suggest that *mcr-1*–producing *E. coli* can colonize companion animals and be transferred between companion animals and humans. The findings also suggest that, in addition to food animals and humans, companion animals can serve as a reservoir of colistin-resistant *E. coli*, adding another layer of complexity to the rapidly evolving epidemiology of plasmid-mediated colistin resistance in the community.

Technical Appendix Pulsed-field gel electrophoresis analysis of 9 *mcr-1*–producing *Escherichia coli* isolates from companion animals and human patients, Guangzhou, China.
