# Supplementary material for: Possible Transmission of mcr-1–Harboring Escherichia coli between Companion Animals and Human
Source: Emerg Infect Dis. 2016 Sep;22(9):1679–81. doi: 10.3201/eid2209.160464 (PMC4994340; doi:10.3201/eid2209.160464)
Supplement: Technical Appendix — Pulsed-field gel electrophoresis analysis of 9 mcr-1–producing Escherichia coli isolates from companion animals and human patients, Guangzhou, China. [file 16-0464-Techapp-s1.pdf]

Article DOI: http://dx.doi.org/10.3201/eid2209.160464

Possible Transmission of *mcr-1*–Harboring *Escherichia coli* between Companion Animals and Human

Technical Appendix

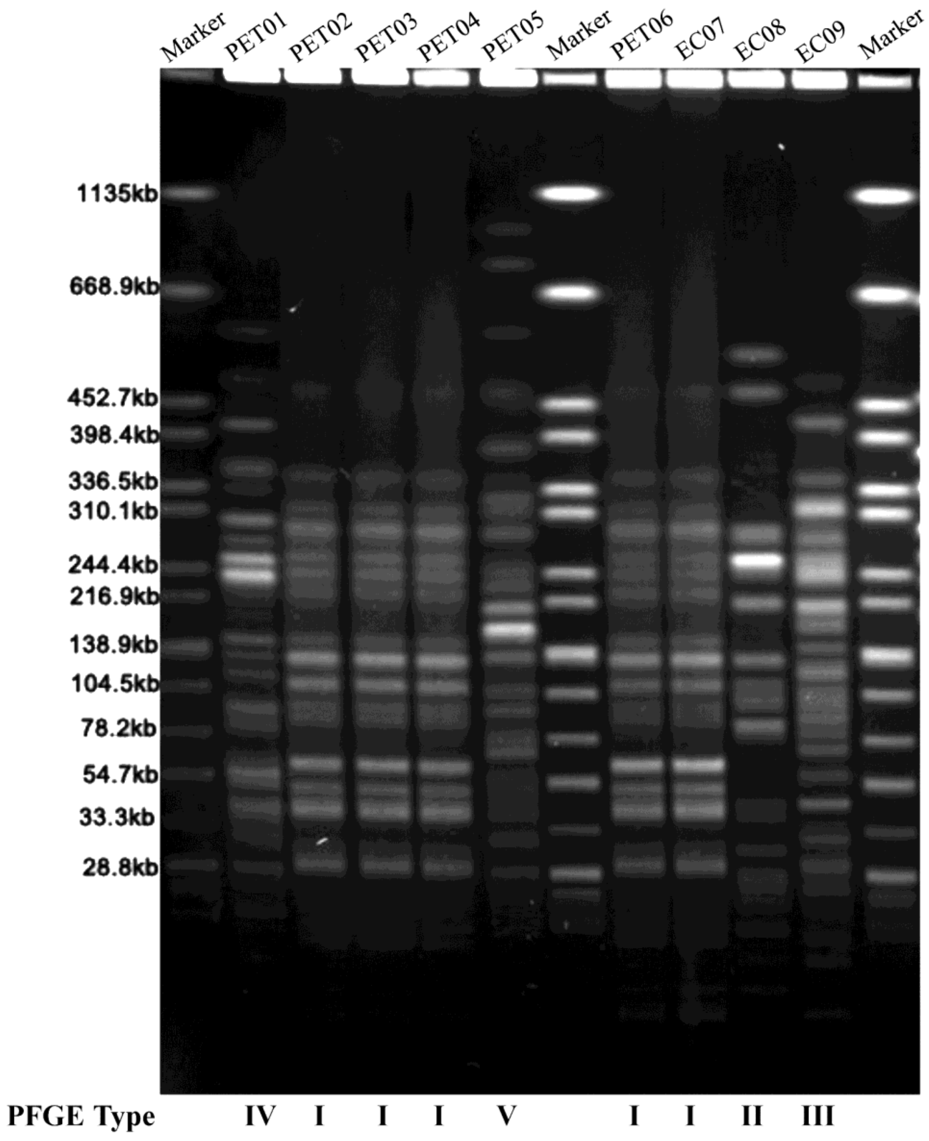

Technical Appendix Figure. Pulsed-field gel electrophoresis analysis of 9 *mcr-1*–producing *Escherichia coli* isolates from companion animals and human patients, Guangzhou, China.
